# Evaluating Improvement in the Documentation of Patient Progress Notes Through the BSOAP Framework: A Clinical Audit

**DOI:** 10.7759/cureus.89980

**Published:** 2025-08-13

**Authors:** Basil Hamed, Imam Mohamed, Raghad Elsayed, Ahmed Ibrahim, Mariam Mohamed, Namarig Ahmed, Arowa Abdalraheem, Esra Ali, Abdalftah Osman, Sara Saeed, Samah Ibrahim, Ahmed Awad

**Affiliations:** 1 Pediatrics, National Ribat University, Khartoum, SDN; 2 Department of Pediatrics, Dongola Specialized Hospital, Dongola, SDN; 3 General Practice, Altababa Advanced Training Center, Khartoum, SDN

**Keywords:** clinical audit system, medical records, patient’s safety, progress notes, quality improvement (qi)

## Abstract

Background

Medical documentation is defined as the manual or electronic writing of health-related information of specific patients. It is a crucial process in patient care that ensures transparency, safety, and continuity of care. This audit aims to evaluate and improve the practice of progress notes documentation using the BSOAP (Background, Subjective, Objective, Assessment, Plan) framework.

Methodology

A retrospective-prospective, three-cycle clinical audit was conducted between March and November 2024 in the pediatric department at Dongola Specialized Hospital. A total of 511 morning follow-ups were evaluated. Intervention included local presentation after each cycle, small group discussion, visual reminders, and supervision.

Results

According to the component, the least documented was the plan. The least documented parameter was discussion and patient education, followed by new investigation requests and review of relevant systems. The most documented parameter was vital signs, followed by patient age. The second and third cycles showed improvement in all components, with higher improvement noted between the second and third cycles. All progress notes in the second and third cycles used a BSOAP-based approach.

Conclusions

Implementation of the BSOAP framework as a structured tool, combined with targeted training and supervision, led to notable improvement in documentation.

## Introduction

Medical documentation refers to the manual or electronic recording of patient health information. It includes, but is not limited to, admission and progress sheets, discharge summaries, and operation sheets [1]. Documentation and record-keeping are crucial processes in patient care that ensure transparency, safety, and continuity of care, in addition to accountability. Further, it is important for scientific research, resource allocation, and auditing [2,3].

Due to the growing complexity of the clinical information across various care settings, the documentation process has become more challenging for junior doctors when writing follow-up progress notes, which can contribute to unfavorable outcomes, including an increased rate of readmission, treatment delay, and improper management and/or diagnosis [2,4,5]. For these reasons, evaluation and auditing of patient notes are essential for reducing medical errors and ensuring adequate service. This also ensures that medical records are accurate, readable, and accessible [2,6].

The SOAP (Subjective, Objective, Assessment, and Plan) note is a well-known acronym invented by Weed [7]. It is a systematic, standardized tool that represents both a cognitive aid and an index for retrieving medical information. The validity and usability of the SOAP checklist have been studied by different healthcare providers, including doctors, nurses, and pharmacists [8,9]. The SOAP progression note is written periodically after each assessment and is considered a legal document and a method of communication between physicians and other healthcare workers. Furthermore, the SOAP progression note is a valuable resource for third parties such as insurance companies and researchers [10].

The subjective section of SOAP reflects the personal view of the presenting complaint, including the patient’s chief complaint, history of present illness, history (including past medical history, surgical history, social history, and family history), review of systems, and current medications and allergies. The objective section includes vital signs, examination findings, and relevant investigations [8,11].

The assessment section aims to use information gained from the subjective and objective sections to formulate and synthesize problem lists and differential diagnoses, which are arranged in order of importance and probability, respectively [8,11]. This is particularly important in pediatric patients, as they experience relatively rapid changes in their status compared to the older population. BSOAP note is an extension of SOAP, where *B* stands for background information. The plan section provides specific actions needed for managing problems and narrowing differential diagnosis that includes medication, specific tests, specialist referrals, and patient education.

This audit aims to evaluate documentation in progress notes in terms of the quality, completeness, and consistency, in addition to identifying the pattern of incomplete entries. It seeks to implement improvement using a well-known SOAP sheet [8,11].

## Materials and methods

A retrospective-prospective study was conducted between March and November 2024 in the Pediatric Department of Dongola Specialized Hospital. The study was approved by the Ethical Review Committee, the Pediatric Department at Dongola Specialized Hospital (protocol number: DSH/IRB/2024/001; dated March 25, 2024). The privacy and confidentiality of collected patient information were ensured. In the first cycle, 109 patients’ files were evaluated from March 1, 2024, to March 31, 2024. Evaluation of the file included all written morning follow-up notes (a total of 226) of those patients.

The second cycle was conducted between July 1, 2024, to July 31, 2024, and included 37 files (124 morning follow-ups). The last cycle targeted 65 files (161 morning follow-ups) from October 20, 2024, to November 20, 2024. The evaluated progress notes were written by house officers. Data were collected electronically using an online checklist and analyzed using SPSS version 26.0 (IBM Corp., Armonk, NY, USA). Results were presented as tables and figures and described as frequencies and percentages. Figures were constructed using Microsoft Excel 2010.

A local presentation was conducted after collecting and analyzing the data of the first cycle. The presentation contained the result of the first cycle, elaborated on the importance of medical documentation, and introduced the BSOAP mnemonic as an effective tool for documentation. The presentation was attended by the house officer, medical officers, and registrars, and followed by a small group discussion about what should be done to improve the practice. All attending staff members were trained in effective methods of documentation using the BSOAP template. A poster illustrating the usage of the BSOAP mnemonic was distributed across the hospital.

We conducted a second presentation, which highlighted the results of the first cycle and specific defects in the documentation. The meeting discussed a standard structured template for progress notes to replace the formal progress note, which was approved by the hospital director. Brief messages illustrating the importance of the BSOAP acronym and how to write effective progress notes were sent regularly, directly to the house officers. Direct supervision of house officers by medical officers was recommended. In the last presentation, the results were discussed with an emphasis on the importance of continuous adherence to standard practice and regular reevaluation.

Audit standards

All follow-up sheets needed to contain 18 parameters, which were based on the National Institute for Health and Care Excellence (NICE) requirements [8] (Table 1). Parameters were evaluated in all progress notes and documented as done or not done. Subsequently, the completeness of each BSOAP component was evaluated as complete (all parameters were documented), partially incomplete (more than 50% of parameters were documented), and not complete (less than 50% were documented).

**Table 1:** Audit standard.

Parameters	Target percentages
Background	
Patient age	100%
Patient gender	100%
Subjective	
Chief complaints	100%
Duration of complaint	100%
Past medical history	100%
Diagnosis	100%
Previous complaint progression	100%
Analysis of new complaints	100%
Review of relevant systems as expected for the disease	100%
Objective	
Vital sign	100%
Physical examination	100%
Investigation	100%
Problem list in a chronological manner	100%
Patient progress	100%
Plan	
Treatment needed, such as medication or a procedure	100%
New investigation request	100%
Patient advice and education	100%
Planned follow-up	100%

## Results

**Figure 1:**
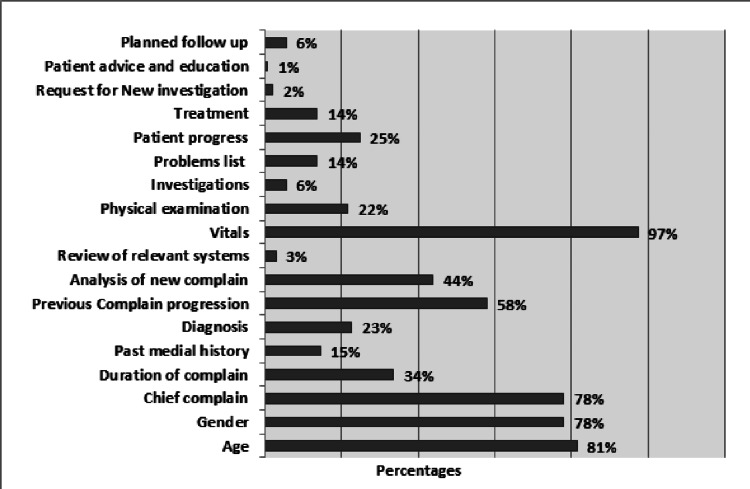
Baseline performance according to specific components.

The audit included a total of 211 patient files (511 progress notes) in three cycles. The first cycle included 226 written morning follow-ups. Upon evaluation of progress notes against the standard, all notes had at least one deficiency in one or more components of the SOAP format. Among all components, the least documented was the plan (92.5% incomplete and 7.5% partially complete) (Table 2). The least documented was discussion and patient education in the plan section (documented in 0.9% of files), followed by the request for new investigation (2.2%) and the review of relevant systems (3.1%). The most consistently documented item was vital signs (97%), followed by patient age (81%) (Figure 1).

**Table 2:** Baseline document completeness according to section.

Section	Incomplete frequency (%)	Partially incomplete frequency (%)	Complete frequency (%)
Background	60 (26.5%)	158 (69.9%)	8 (3.5%)
Subjective	145 (64.2%)	77 (34.1%)	4 (1.8%)
Objective	160 (70.8%)	59 (26.1%)	7 (3.1%)
Assessment	161 (71.2%)	42 (18.6%)	23 (10.2%)
Plan	209 (92.5%)	17 (7.5%)	0 (0%)

The second and third cycles showed improvements in all components, with greater gains observed between the second and third cycles. All progress notes in the second and third cycles used a BSOAP-based approach (Table 3).

**Table 3:** Application of assessment parameters.

	First cycle percentages	Second cycle percentages	Improvement percentages	Third cycle percentages	Improvement percentages
Background					
Age	81.4%	86.9%	+6%	99%	+12%
Gender	77.9%	84.3%	+6%	95%	+11%
Subjective					
Chief complaint	77.9%	85.4%	+7%	99%	+14%
Duration of complaint	33.6%	49.4%	+15%	93%	+44%
Past medical history	14.6%	30%	+15%	86%	+56%
Diagnosis	22.6%	46.3%	+23%	94%	+48%
Previous Complaint progression	58.0%	70.6%	+13%	97%	+26%
Analysis of new complaints	43.8%	60.3%	+16%	94%	+34%
Review of relevant systems	3.1%	17.1%	+14%	88%	+71%
Objective					
Vitals	97.3%	98%	+1%	99%	+1%
Physical examination	21.7%	34.7%	+13%	90%	+55%
Investigations	5.8%	21.1%	+15%	67%	+46%
Assessment					
Problem list in a chronological manner	13.7%	31.7%	+18%	63%	+31%
Patient progress	24.8%	45.4%	+20%	88%	+43%
Plan					
Treatment	13.7%	38.3%	+24%	94%	+56%
Request for new investigation	2.2%	28.6%	+27%	69%	+40%
Patient advice and education	0.9%	14.3%	+13%	57%	+43%
Planned follow-up	5.8%	22%	+16%	66%	+44%

**Figure 2:**
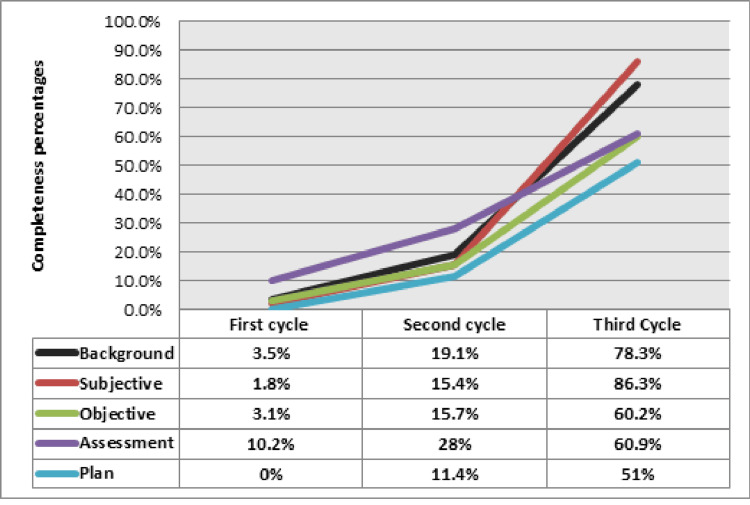
Percentages of completeness of progress notes.

The most documented points in the third cycle were age, chief complaint, and vital signs (99%). The least documented points were patient advice and education (57%), problem list (63%), planned follow-up (66%), and investigation (67%). According to the component-level analysis, the subjective section was fully documented in 86%, followed by the background (78%), assessment (61%), objective (60%), and plan (51%) (Figure 2). Table 4 illustrates the results of the third cycle. The lowest documentation was observed in the plan section, with 27% marked as incomplete and 22% as partially incomplete.

**Table 4:** Completeness of progress notes by section (third cycle).

Third cycle	Background percentages	Subjective percentages	Objective percentages	Assessment percentages	Plan percentages
Complete	78%	86%	60%	61%	51%
Partially incomplete	21%	9%	35%	30%	22%
Incomplete	1%	5%	4%	9%	27%

## Discussion

Documentation of follow-up notes plays a crucial role in tracking patient progress, changes in the problem list, and any emerging issues that may necessitate timely intervention, especially in the pediatric population. Poor documentation of progress notes may result in decreased quality of the delivered care and patient satisfaction.

Irrespective of the evolving electronic health record system, paper-based health records remain the standard method for documentation in Sudan. The admission file contains a uniform printed template for baseline admission history, examination, investigation, and admission plan (filled out by medical officers), followed by blank pages for documentation of progress notes (filled out by house officers). The unavailability of a clear proforma for progression notes led to high variability between entries, with the omission of critical parameters (patient education was omitted in our study). Our audit aimed to adopt the BSOAP framework as an effective method for documentation.

Multiple quality improvement projects have been conducted in developing countries and have shown good outcomes, which contradicts the common belief of non-applicability of audit in these countries [12]. In 2024, Awad et al. performed an audit that aimed to improve the quality of documentation in Managil Teaching Hospital, Sudan. The study mainly focused on the quality of the whole patient file, in contrast to our study, which mainly focused on the progress notes, which we believe have a higher degree of deficiency than baseline admission data due to the aforementioned reasons. The second main difference is that we used a well-structured BSOAP framework for the evaluation of documentation and training staff members, which has proven to be simple and effective in low-resource settings and provides specific details about patients’ progress [13,14]. In 2022, Gasoma also performed a two-cycle clinical audit to assess documentation practice in surgical patients in Ribat University Hospital, Sudan. He evaluated the documentation using local guidelines that were formulated by the Sudan Medical Council. He reported an average increase in compliance by 32% after intervention [15].

The least documented parameters in our study were patient education (0% in the first cycle), requested investigation, systematic review, and key investigation results. Even though all patient-requested laboratory results are usually attached to the patient file (which may explain the low documentation rate), writing important laboratory findings is critical and allows easy identification of abnormalities without checking all laboratory results.

After performing the first intervention, documentation of progress notes changed to the BSOAP framework-based documentation in all observed notes. Despite this substantial shift in documentation style, there were still defects in parameters within the five main sections. Percentages of the fully completed section in the second cycle ranged from 11% to 28%, with the plan section showing the lowest percentage of completeness (Figure 2).

This finding highlights some degree of improvement between the first and second cycle; however, it is less pronounced than the changes between the second and third cycle. This may reflect the high effectiveness of the second intervention, which involves active supervision by a medical officer and regular brief illustration of BSOAP direct messages compared to passive training and posters. This approach can be considered for future interventions.

The third cycle showed high percentages of completeness (background and subjective sections were fully documented with all parameters in 78% and 86% of files, respectively). Objective and assessment sections were fully documented in approximately 60% of files in both sections, and incomplete sections were only 4% and 9%, respectively.

Despite substantial improvement in the documentation of the plan section (no file was fully documented in the first cycle), the complete plan section in the third cycle was still 51%, which still needs further improvement. The plan component may remain poorly documented, as planned investigations, patient advice, and follow-up instructions are often overlooked during documentation. A fixed, pre-printed SOAP template may be required to optimize the practice. Additionally, continuous audit-feedback cycles and targeted staff training are required to sustain improvements.

Limitations

This audit was conducted in a single department within one hospital, with a short follow-up period and no control group, which may limit the generalizability of the findings in other regions, specialties, or documentation systems.

## Conclusions

A progress note is a critical document for assessing a patient’s current status. It facilitates efficient communication among healthcare providers and aids decision-making. It also reduces the risk of errors and serves as a legal document. Our study highlights significant defects in the documentation of progress notes, which are primarily due to the absence of a standardized template. Implementation of the BSOAP framework as a structured tool, combined with targeted training and supervision, led to notable improvement in documentation.
